# PAR-2, IL-4R, TGF-β and TNF-α in bronchoalveolar lavage distinguishes extrinsic allergic alveolitis from sarcoidosis

**DOI:** 10.3892/etm.2014.1776

**Published:** 2014-06-11

**Authors:** RADOSLAV MATĚJ, MAGDALENA SMĚTÁKOVÁ, MARTINA VAŠÁKOVÁ, JANA NOVÁKOVÁ, MARTINA ŠTERCLOVÁ, JAROMÍR KUKAL, TOMÁŠ OLEJÁR

**Affiliations:** 1 Department of Pathology and Molecular Medicine, Thomayer Hospital, Prague 140 59, Czech Republic; 2 Department of Pathology, Third Faculty of Medicine, Charles University, Prague 100 00, Czech Republic; 3 Department of Respiratory Medicine, Thomayer Hospital, Prague 140 59, Czech Republic; 4 Faculty of Nuclear Sciences and Physical Engineering, Czech Technical University, Prague 115 19, Czech Republic; 5 Department No. 75, Institute of Physiology, Academy of Sciences of the Czech Republic, Prague 142 20, Czech Republic

**Keywords:** sarcoidosis, extrinsic allergic alveolitis, interleukin-4 receptor, transforming growth factor-β, tumor necrosis factor-α, proteinase-activated receptor-2

## Abstract

Sarcoidosis (SARC) and extrinsic allergic alveolitis (EAA) share certain markers, making a differential diagnosis difficult even with histopathological investigation. In lung tissue, proteinase-activated receptor-2 (PAR-2) is primarily investigated with regard to epithelial and inflammatory perspectives. Varying levels of certain chemokines can be a useful tool for distinguishing EAA and SARC. Thus, in the present study, differences in the levels of transforming growth factor (TGF)-β1, tumor necrosis factor (TNF)-α, interleukin-4 receptor (IL-4R) and PAR-2 in bronchoalveolar lavage fluid (BALF) were compared, using an ELISA method, between 14 patients with EAA and six patients with SARC. Statistically significant higher levels of IL-4R, PAR-2 and the PAR-2/TGF-β1 and PAR-2/TNF-α ratios were observed in EAA patients as compared with SARC patients. Furthermore, the ratios of TNF-α/total protein, TGF-β1/PAR-2 and TNF-α/PAR-2 were significantly lower in EAA patients than in SARC patients. The results indicated a higher detection of PAR-2 in EAA samples in association with TNF-α and TGF-β levels. As EAA and PAR-2 in parallel belong to the Th2-mediated pathway, the results significantly indicated an association between this receptor and etiology. In addition, the results indicated that SARC is predominantly a granulomatous inflammatory disease, thus, higher levels of TNF-α are observed. Therefore, the detection of PAR-2 and investigated chemokines in BALF may serve as a useful tool in the differential diagnosis between EAA and SARC.

## Introduction

**Figure 1:**
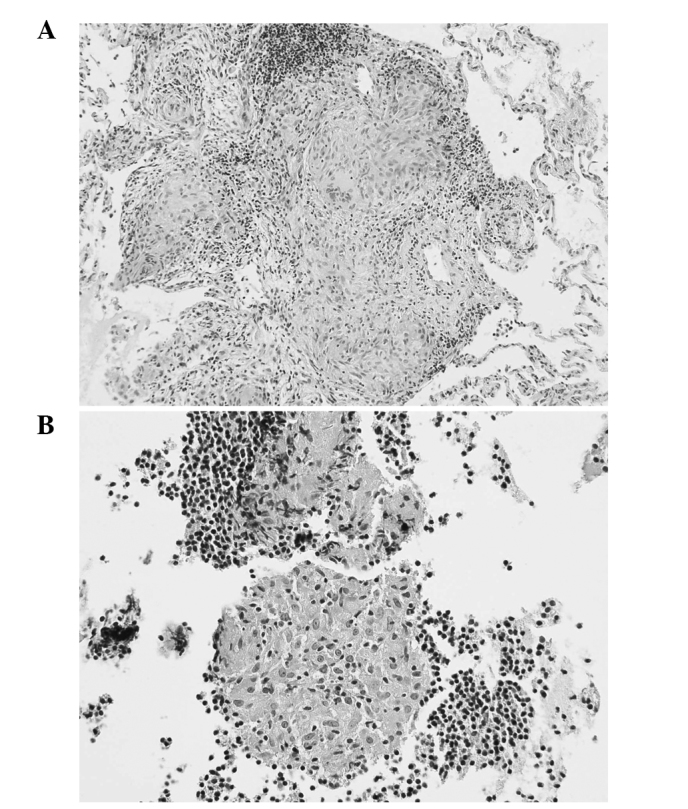
(A) Histopathological image of an epithelioid granuloma of sarcoid type in the transbronchial biopsy specimen of the peribronchial lung parenchyma, compatible with a diagnosis of pulmonary SARC (HE; magnification, ×200). (B) Epithelioid granuloma of sarcoid type formation in the mediastinal lymphatic node in an EBUS-TBNA specimen, compatible with lymphatic node involvement by SARC (HE; magnification, ×400). SARC, sarcoidosis; HE, hematoxylin and eosin; EBUS-TBNA, endobronchial ultrasound-guided transbronchial needle aspiration.

In sarcoidosis (SARC), a Th1 immune reaction predominates (1), while in extrinsic allergic alveolitis (EAA), Th2 immunity, associated with an allergen exposure, is primarily involved (2,3). Despite having a different etiopathogenesis, morphologically, the two etiologies share certain markers, including granulomas, interstitial lymphocyte infiltration and fibrosis, making a differential diagnosis difficult even with histopathological investigation. While SARC can be diagnosed by an endoscopical transbronchial biopsy of the lung parenchyma and an endobronchial ultrasound-guided transbronchial needle aspiration of the mediastinal lymphatic nodes (Fig. 1; sample from subject belonging to target SARC cohort), histopathological confirmation of EAA often requires a surgical biopsy.

**Figure 2:**
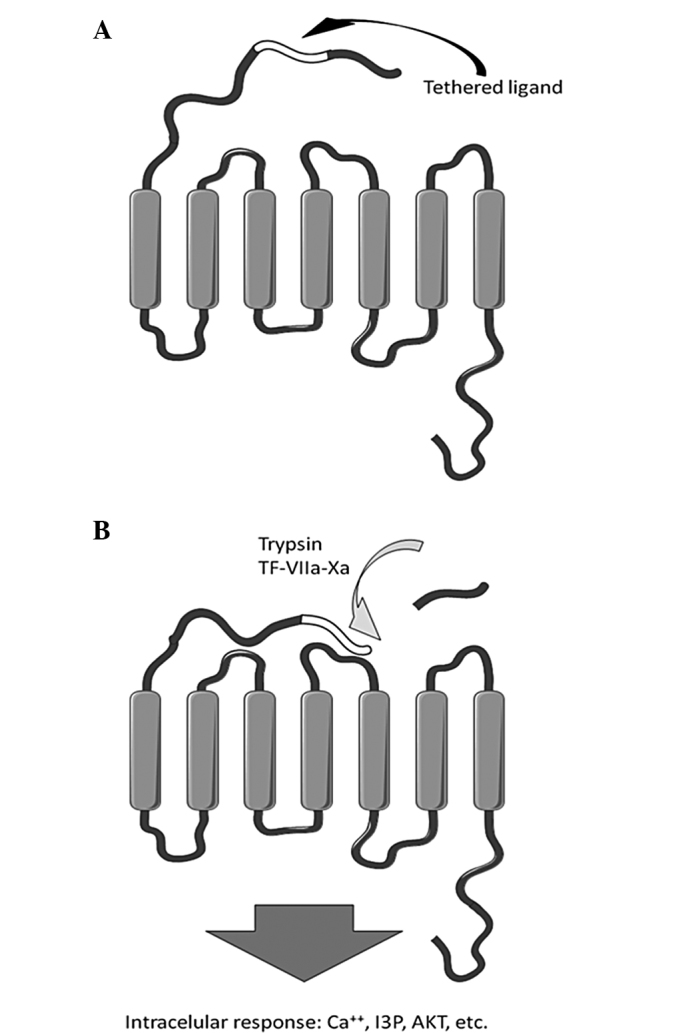
Schematic conformational status (A) prior to and (B) following PAR-2 activation. Following the specific proteolytic cleavage of the receptor, the new N-terminal is presented to the transmembrane domains as the tethered ligand. Receptor activation leads to various intracellular responses (6). The figure was composed using Servier Powerpoint image bank: www.servier.com. PAR-2, proteinase-activated receptor-2.

Proteinase-activated receptors (PARs) are ubiquitous surface molecules directly interconnecting immunity and coagulation (4,5). PARs belong to a family of G protein-coupled receptors, activated by tethered ligand sequences within the N-terminal, that are accessible following site specific cleavage of the protein (6,7), as demonstrated in Fig. 2. PAR-1, PAR-3 and PAR-4, among others, are activated by the plasma coagulation factor, thrombin, while PAR-2 is activated by trypsin, tryptase and a complex of coagulation factors, including tissue factor/VIIa/Xa (8).

In lung tissue, PAR-2 is predominantly investigated with regard to epithelial and inflammatory perspectives. In the lungs, PAR-2 is also a target for mast cell tryptase, *Alternaria alternata* serine proteinases and fungal asthmagens (9).

Associations among PAR-2, interleukin-4 receptor (IL-4R), transforming growth factor (TGF)-β and thymic stromal lymphoprotein (TSLP) have already been investigated in bronchial asthma, chronic obstructive pulmonary disease (COPD) (10) and idiopathic lung fibrosis (11). PAR-2 has been shown to induce (12) TSLP, which is a known inducer of Th2 naive T cell differentiation via dendritic cell maturation (13,14). In addition, TSLP and *Alternaria alternata*-induced production in bronchial epithelial cells via PAR-2 activation, is synergically enhanced by IL-4 (12). There are no available data directly connecting PAR-2, IL-4 and TSLP in the involvement of alveolar epithelium in EAA or SARC, however, there is a high probability that this autocrine/paracrine loop may contribute to the upregulation of IL-4 in these nosologies (15). There is marked evidence that a number of additional enzymes and their receptors are involved in these complex processes; which raises the question of whether their role is primary or unspecific and secondarily-induced. However, it has recently been reported that TGF-β stimulates PAR-2 production in human lung fibroblasts (16), which demonstrates its role in the pathophysiology of idiopathic pulmonary fibrosis. The evidence indicates that TGF-β generally induces PAR-2 overexpression, regulating fibrosis and scar formation (17). However, higher levels of TGF-β have been observed in bronchial asthma and COPD patients (18), as well as in SARC (19) and EAA (20). In the two diseases, tumor necrosis factor (TNF)-α also plays an important proinflammatory role; alveolar macrophages are the main source of this cytokine (21). In an *in vitro* model of alternatively activated macrophages, lipopolysacharide-induced PAR-2 activation suppressed the mRNA expression of proinflammatory cytokines, including TNF-α (22), with a feedback loop to the previously mentioned IL-4. By contrast, PAR-2 activation together with parallel ovalbumin exposure leads to TNF-dependent allergic sensitization (23).

## Materials and methods

### Subjects

A total of 20 patients were enrolled in the study. All the individuals were outpatients of the Department of Respiratory Medicine at Thomayer Hospital (Prague, Czech Republic). The patients underwent a bronchoscopic investigation as part of a differential diagnosis for interstitial lung disease with bronchoalveolar lavage fluid (BALF) analysis.

In total, six patients (mean age, 44.7 years; male, 4; female, 2) were diagnosed with SARC, according to the American Thoracic Society/European Respiratory Society/World Association of Sarcoidosis and Granulomatous Disorders statement on SARC (24). The diagnosis was based on patient history, clinical symptoms, standard chest radiography, high resolution computed tomography (HRCT) and laboratory tests (serum angiotensin converting enzyme, calcemia and calciuria). All the patients underwent a transbronchial biopsy, transbronchial lymph node puncture or video-thoracoscopic lung biopsy with histopathological evidence supporting the diagnosis of SARC. For histopatology, 10% formalin-fixed, paraffin-emebeded biopsy samples were cut to microscopic tissue slices, 5 μm thick, xylene-deparaffined, ethanol-rehydrated and according to standard protocol stained with hematoxylin and eosin (HE).

The diagnosis of EAA in 14 patients (mean age, 56.2 years; male, 7; female, 7) was based on the history of exposure to a suspect antigen, the typical clinical course, HRCT radiological observations compatible with EAA, the BALF cell count and levels of specific IgG to the suspect antigen.

All the patients signed an informed consent form prior to the start of the study. The study design and the informed consent form were approved by the Central Ethical Committee of the Thomayer Hospital and the Institute for Clinical and Experimental Medicine (Prague, Czech Republic). Additionally, all data were analyzed with respect for patient privacy.

### BALF collection

BALF collection was performed during the fiber-optic bronchoscopy under local anesthesia. Five 50-ml fractions of lukewarm saline were instilled into the segmental section of the middle lobe where the bronchoscope was wedged. The fluid was retrieved using syringe suction and mixed in a container prior to being divided for further investigation. The sample was determined to be valid if the recovery was >20 ml per fraction and a significant mixture of polymorphic bronchial epithelial cells was not identified.

### ELISA

Concentrations of particular analytes in the BALF were determined using the ELISA method. The kits were purchased from Uscn Life Science, Inc. (Wuhan, China). Reactions were conducted in microtiter plate wells that had been precoated with monoclonal antibodies (mAb) specific for the examined analyte (IL-4R, E92031Hu; PAR-2, E90852Hu; TGF-β1, E90124Hu; TNF-α, E90133Hu). A labeled polyclonal antibody was intended to bind to the mAb-analyte complex. Following the reaction with the substrate solution, the process was terminated. The colored products that were formed were measured with a vertical colorimeter (EL800; Bio-Tek Instruments, Inc., Winooski, VT, USA) and the concentration of the analyte in the samples was determined using a standard curve.

### Statistical analysis

Data were collected from the two groups consisting of 14 EAA patients and six SARC patients. The differences in 25 basic and derived characteristics were analyzed with a standard two-sample, two-sided t-test, where P<0.05 was considered to indicate a statistically significant difference. In cases of multiple testing, the Bonferroni correction was used as required. In addition, the false discovery rate (FDR) methodology was used for the 25 independent tests and a corrected critical level of 0.004 [2 × (0.05/25)] was calculated, which resulted in only two significant differences. All the calculations were performed using MATLAB 7.8.0 Statistical Toolbox (Mathworks Inc., Natick, MA, USA, 2009).

## Results

### Higher parameters in EAA

**Figure 3:**
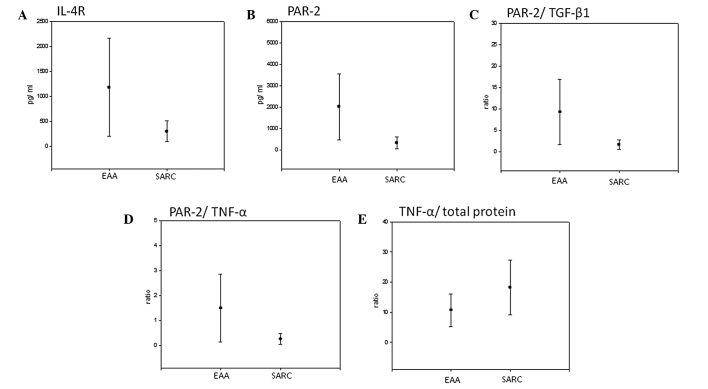
Statistically significant higher levels (P<0.05) of (A) IL-4R (1182.7 vs. 302.7 pg/ml; P=0.046), (B) PAR-2 (2009.4 vs. 329.5 pg/ml; P=0.018), (C) PAR-2/TGF-β1 ratio (9.29 vs. 1.61; P=0.026) and (D) PAR-2/TNF-α ratio (1.5 vs. 0.26; P=0.042) were identified in EAA patients as compared with SARC patients. (E) The ratio of TNF-α/total protein was significantly lower in EAA patients than in SARC patients (10.64 vs. 18.24; P=0.032). IL-4R, interleukin-4 receptor; PAR-2, proteinase-activated receptor-2; TGF, transforming growth factor; TNF, tumor necrosis factor; EAA, extrinsic allergic alveolitis; SARC, sarcoidosis

Statistically significant higher levels of IL-4R (1182.7 pg/ml vs. 302.7 pg/ml; P=0.046; Fig. 3A), PAR-2 (2009.4 pg/ml vs. 329.5 pg/ml; P=0.018; Fig. 3B) and the PAR-2/TGF-β1 (9.29 vs. 1.61; P=0.026, Fig. 3C) and PAR-2/TNF-α ratios (1.5 vs. 0.26; P=0.042; Fig. 3D) were identified in EAA patients as compared with SARC patients. All the tested characteristics, average values per group and respective P-values are shown in Table I.

### Higher parameters in SARC

**Figure 4:**
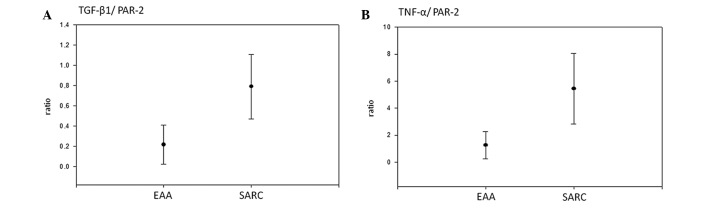
(A) TGF-β1/PAR-2 ratio derived from individual results using the ELISA method. The ratio (0.217 vs. 0.791; P=0.0000923) was significantly lower in EAA cases and the statistically significant difference complied with a FDR-adjusted critical level of 0.004. (B) TNF-α/PAR-2 ratio derived from individual results using the ELISA method. The ratio (1.26 vs. 5.45; P=0.0000503) was significantly lower in cases of EAA and the statistically significant difference complied with a FDR-adjusted critical level of 0.004. TGF, transforming growth factor; PAR-2, proteinase-activated receptor-2; EAA, extrinsic allergic alveolitis; FDR, false discovery rate; TNF, tumor necrosis factor.

By contrast, the ratio of TNF-α/total protein was significantly lower in EAA patients than in SARC patients (10.64 vs. 18.24; P=0.032; Fig. 3E). Furthermore, the ratios of TGF-β1/PAR-2 (0.217 vs. 0.791; P=0.0000923) and TNF-α/PAR-2 (1.26 vs. 5.45; P=0.0000503) were significantly lower in EAA cases with regard to the FDR methodology, as shown in Fig. 4.

## Discussion

In a pilot immunoassay study, statistically significant higher levels of PAR-2, IL-4R and PAR-2/TGF-β and PAR-2/TNF-α ratios were identified in BALF samples from EAA patients. In addition, following FDR adjustment, statistically significant higher ratios of TGF-β/PAR-2 and TNF-α/PAR-2 were observed in BALF samples from SARC patients.

The immunoassay differences in the level of PAR-2 and the associated TGF-β and TNF-α ratios in BALF may have resulted from a different ratio between specific (activation) and non-specific (shedding induced inactivation) cleavage of the receptor. A variety of enzymes, including tryptase, elastase, human airway trypsin-like protease, cathepsin G and matrix metaloproteinases (MMPs), are released from neutrophils, mast cells, alveolar macrophages and airway epithelium, which results in rate dependent activation/inactivation effects on PAR-2 (25). In addition, membrane-bound proteinases, such as proteinase 3 that is involved in activation and inactivation modes of PAR-2, was also expressed in neutrophils and alveolar macrophages clearing TNF-α, which is more common in interstitial pneumonitis than in SARC (26). BALF and membrane proteinases may, in a rate dependent manner, influence (in parallel) membrane receptors, such as PARs, and soluble cytokines, including TGF-β and TNF-α. By contrast, PAR-2 induces the mRNA expression of MMP-9 (27), and MMP-9 induces TGF-β production in airway epithelial cells (28). Higher levels of TGF-β were detected in BALF samples from lung regions, indicating increased EAA and SARC activity, as estimated by the HRCT score (19).

To date, it is not clear whether a specific or a non-specific chain from the N-terminal has been detected, however, the results of the present study indicate that in EAA, substantially more PAR-2 terminals are released. The results demonstrate a higher detection of PAR-2 in EAA samples, which is in association with levels of TNF-α and TGF-β. As EAA and PAR-2, in parallel, belong to the Th2-mediated pathway (29), the results strongly indicate an association between this receptor and etiology. The results of the current study also indicate that SARC is predominantly a granulomatous inflammatory disease, thus, higher levels of TNF-α are observed (30). The EAA subjects in the present study were predominantly elderly, with a sub-acute or chronic course of the disease. Thus, inclination toward fibrosis and correlation with higher PAR-2 levels is expected in association with repeated, long term exposure to different proteolytic enzymes (31) despite to its specific and non-specific cleavage. A previous study investigated the dissociated gene and protein expression levels of PAR-2 in cultured alveolar macrophages from smokers and healthy subjects (15), and raised the question of whether the presence of surface protein in the BALF may also be investigated as a possible biomarker for the transformation of EAA, or an additional interstitial process, into a more chronic fibrosing course.

In conclusion, the detection of PAR-2 and specific chemokines in the BALF may serve as a useful tool in the differential diagnosis between EAA and SARC during routinely used bronchoscopical investigation. This method can prevent more invasive surgical pulmonary biopsy verification, particularly in cases of EAA.

## Figures and Tables

**Table I:** Statistical analysis of the basic and derived characteristics (n=25).

Characteristic	Average EAA value (pg/ml)	Average SARC value (pg/ml)	P-value
Total protein	209.4286	80.5667	0.16239
IL-4R	1182.7071	302.7167	0.045507^a^
PAR-2	2009.4143	329.5333	0.017622^a^
TGF-β	227.8143	194.0333	0.40364
TNF-α	1381.9357	1292.2333	0.6782
Total protein/IL-4R	1.2195	0.70294	0.57665
Total protein/PAR-2	0.30052	0.32913	0.92454
Total protein/TGF-β	1.0184	0.40749	0.18103
Total protein/TNF-α	0.15603	0.065155	0.22735
IL-4R/total protein	8.1408	4.6233	0.26063
IL-4R/PAR-2	1.2863	1.6243	0.73537
IL-4R/TGF-β	5.9655	1.7875	0.080645
IL-4R/TNF-A	0.92805	0.30629	0.092826
PAR-2/total protein	17.8294	4.0955	0.14669
PAR-2/IL-4R	8.1433	2.7456	0.25928
PAR-2/TGF-β	9.292	1.6143	0.025914^a^
PAR-2/TNF-α	1.5019	0.25849	0.041555^a^
TGF-β/total protein	1.9687	2.5616	0.38217
TGF-β/IL-4R	1.7496	1.7226	0.98542
TGF-β/PAR-2	0.217	0.79093	0.00009231^b^
TGF-β/TNF-α	0.17341	0.15672	0.56945
TNF-α/total protein	10.6382	18.2398	0.032419^a^
TNF-α/IL-4R	10.0243	12.3861	0.77667
TNF-α/PAR-2	1.2594	5.4468	0.000050292^b^
TNF-α/TGF-β	6.5795	6.947	0.76699

a P<0.05 and

b P<0.004 (FDR adjusted critical level).

FDR, false discovery rate; EAA, extrinsic allergic alveolitis; SARC, sarcoidosis; IL-4R, interleukin-4 receptor; PAR-2, proteinase-activated receptor-2; TGF, transforming growth factor; TNF, tumor necrosis factor.
